# Unraveling multipredator impacts in salmon‐bearing rivers using quantitative DNA metabarcoding

**DOI:** 10.1002/eap.70158

**Published:** 2025-12-18

**Authors:** John J. Winkowski, Lisa M. Crosson, Julian D. Olden

**Affiliations:** ^1^ Washington Department of Fish and Wildlife Olympia Washington USA; ^2^ School of Aquatic and Fishery Sciences University of Washington Seattle Washington USA

**Keywords:** bass, diet analysis, DNA metabarcoding, intraguild, invasive, multi‐invader, nonnative, predation, salmon

## Abstract

Ecological impacts of invasive species are mounting as their numbers and geographic extent continue to increase. Across extensive parts of their range, Pacific salmon (*Oncorhynchus* spp.) smolts face an expanding gauntlet of nonnative predators during their seaward migration. Adopting multispecies, spatiotemporal perspectives is essential for understanding direct and indirect predation risks and prioritizing management actions seeking to reduce impacts. Using quantitative DNA metabarcoding, we investigated trophic interactions of commonly co‐occurring nonnative and native fish predators of Pacific Northwest, USA, salmon‐bearing rivers, addressing challenges for salmon recovery and questions related to single‐species management. Chinook salmon (*Oncorhynchus tshawytscha*) were frequently consumed by nonnative smallmouth bass (*Micropterus dolomieu*), largemouth bass (*Micropterus salmoides*), rock bass (*Ambloplites rupestris*), and native northern pikeminnow (*Ptychocheilus oregonensis*). Among the focal predators, Chinook salmon were the largest contributors to smallmouth bass diets, ranking as their second most important prey. Chinook salmon consumption peaked during a year of relatively high smolt abundance, low discharge, and warm stream temperatures. The following year, under opposite conditions, Chinook salmon consumption declined, though predation remained disproportionately high in certain mainstem and tributary regions. Native species of conservation concern were frequently consumed by nonnative predators, including imperiled native lamprey (family *Petromyzontidae*). Across space and time, native prickly sculpin (*Cottus asper*) and largescale sucker (*Catostomus macrocheilus*) were generally the highest contributing prey for nonnative predators. Intraguild predation was prevalent, most notably with smallmouth bass as the top prey for northern pikeminnow. Intraguild predation highlights potential risks of compensatory effects when predators are managed in isolation. Our study provides crucial insights into restoring food webs for native species while minimizing the likelihood of compensatory effects and demonstrates the value of quantitative DNA metabarcoding for understanding novel predator assemblages. As ecosystems worldwide face increasing pressures from co‐occurring invasive species, integrating multispecies approaches into management strategies is essential for mitigating impacts and conserving biodiversity.

## INTRODUCTION

The ecological impacts of invasive species have long been recognized, spanning diverse taxonomies, geographies, and levels of biological organization, from genes to ecosystems (Early et al., 2016). Alarming rates of human‐mediated movement of invasive species in recent decades, coupled with ongoing climate change, have only served to further daylight the gap between ecological knowledge and management solutions (Beaury et al., 2020). Among the many emerging persistent challenges, managing multiple sympatric invaders remains notably complex (Courchamp et al., 2003; Doherty et al., 2015). Bridging the knowing–doing gap by integrating multispecies perspectives into invasive species management strategies is a growing priority.

Biological communities hosting multiple invaders often experience different impact regimes compared to systems with a single invader, primarily due to biological interactions that shape their combined effects (Jackson, 2015). Failing to consider these interactions when implementing management strategies can lead to ecological surprises and unintended consequences, often at high remedial costs (Bergstrom et al., 2009; Lurgi et al., 2018; Zavaleta et al., 2001). For instance, sympatric invaders can compete or engage in intraguild predation, suggesting that suppressing or eradicating a species in isolation may trigger the proliferation of equally or more damaging conspecifics (e.g., the “mesopredator release” effect or “competitive release”; Ballari et al., 2016; Ritchie & Johnson, 2009; Tompkins & Veltman, 2006). Moreover, removing invaders in complex systems can impact density‐dependent processes, leading to overcompensation and counterintuitive population booms driven by the “hydra effect” (Abrams, 2009; Grosholz et al., 2021). Trophic niche divergence may enable competing invaders to coexist, effectively broadening their collective impact on the recipient ecological community (Jackson et al., 2014). Conversely, in cases where invasive species do not interact, their impacts may be considered independent, suggesting that management efforts should prioritize the invader with the greatest impact.

Invasive freshwater predators can severely disrupt food webs, altering native species abundance and ecosystem processes (Cucherousset & Olden, 2011; Eby et al., 2006). Widespread historical acclimatization practices and economic motivations to establish recreational fisheries have sparked the intentional or illegal stocking of numerous nonnative predatory gamefishes across the world (Gozlan et al., 2010). While these fishes provide important socioeconomic benefits, their predation impacts on native species have long raised conservation concerns, and in recent decades, prompted management actions globally (McDowall, 2006; Zengeya et al., 2017). In the Pacific Northwest, USA, predation by nonnative gamefish presents a significant threat to salmon (*Oncorhynchus* spp.) population recovery efforts (Sanderson et al., 2009), prompting greater attention towards food web approaches to address their effects (Naiman et al., 2012).

Globally recognized for harmful impacts outside their native range, black basses, including smallmouth bass (*Micropterus dolomieu*) and largemouth bass (*Micropterus salmoides*), are often implicated as primary predators on juvenile Pacific salmon (Carey et al., 2011; Fayram & Sibley, 2000; Fritts & Pearsons, 2004). Other centrarchid species, including rock bass (*Ambloplites rupestris*), remain understudied despite co‐occurring with smallmouth and largemouth bass, and significantly impacting native salmonids and food webs outside their native range (Vander Zanden et al., 1999). Collectively, these nonnative predators also co‐occur with native northern pikeminnow (*Ptychocheilus oregonensis*), which exert strong predation on outmigrating salmon smolts, particularly in heavily modified river reaches of the Columbia River (Beamesderfer et al., 1996; Carey et al., 2012).

Predation impacts on salmon are expected, driven by the opportunistic behaviors of predators and phenological synchrony, as migrating salmon smolts provide a pulsed subsidy coinciding with warming stream temperatures and heightened energy demands during the spring predator spawning period. Consumption of other native species of conservation concern is also likely; for example, growing evidence suggests nonnative fish predation is contributing to regional declines of Pacific lamprey (*Entosphenus tridentatus*; Clemens et al., 2017; Schultz et al., 2017). Each predator's distinct biological traits, including bioenergetic constraints, thermal preferences, and foraging behavior, are likely to result in varying spatiotemporal predation patterns (Petersen & Kitchell, 2001; Webb, 1984). Temporal dietary trends (e.g., interannually and seasonally) may vary in response to abiotic and biotic factors, such as temperature, stream flow, and salmon smolt availability (Durant et al., 2007; Yard et al., 2011). Moreover, predation risk for outmigrating salmon smolts likely varies as they encounter different combinations and densities of predators across heterogeneous riverscapes (Petersen & DeAngelis, 2000; Tiffan et al., 2020). This underscores the need for a spatiotemporal approach to identify predation “hotspots” where resource‐limited managers can prioritize actions (Michel et al., 2020). Moreover, predator interactions, including competition and intraguild predation, likely shape species‐specific and combined predation impacts, influencing the outcomes of single‐species suppression efforts (Rytwinski et al., 2019). Quantifying this information is critical for developing strategies to mitigate predation pressures on imperiled Pacific salmon and other native species, a challenge that resonates broadly across ecosystems with co‐occurring introduced predators.

Integrating knowledge of invasive predator trophic interactions into management strategies is crucial, yet challenging, due to the potential complexity of these interactions. Traditional visual identification of prey in stomach contents is often challenged by rapid digestion rates and unidentifiable prey remains (Legler et al., 2010). Undetected trophic links can lead to the mischaracterization or underestimation of invasive predator impacts, inaccurate food web representations, and the misinformed development of management strategies (Hoare et al., 2007). Molecular‐based diet analyses address these challenges by detecting prey species DNA with high sensitivity and extending the detection period post‐ingestion (Brandl et al., 2016; Dick et al., 2023), thereby leading to a more comprehensive understanding of invasive predator impacts (Brandl et al., 2021). Moreover, high‐throughput sequencing techniques, such as DNA metabarcoding, enable the simultaneous characterization of predator–prey interactions across multiple species, providing detailed insights into animal diets and trophic relationships. Thus, DNA metabarcoding presents new opportunities to reveal complex invasive predator trophic interactions in novel food web assemblages, offering valuable information for multispecies management.

Recent advances in DNA metabarcoding analyses have increased the potential to translate DNA metabarcoding data into accurate quantitative information about the sampled environment (Shelton et al., 2023). Assuming the DNA composition before metabarcoding is representative of the sampled environment, one key challenge in this translation has been bias introduced during the polymerase chain reaction (PCR) amplification process used to generate sequence data (Kelly et al., 2019; Shelton et al., 2016). Species‐specific amplification efficiencies vary with primer sets for specific gene regions (e.g., COI, 12S, or 16S), resulting in biased estimates of the true DNA composition in environmental samples. Recently developed quantitative models address this bias by estimating amplification efficiencies from “mock communities” with known starting DNA proportions and calibrating raw read proportions of environmental samples (Shelton et al., 2023). In the context of diet analyses, these advancements enable the simultaneous characterization of complex trophic links and their relative strengths, providing more robust insights into contemporary food webs, predation impacts, and the integration of this information into management plans (Fisher et al., 2024).

We advance our understanding of the spatiotemporal impacts and trophic interactions of commonly co‐occurring nonnative and native fish predators in Pacific Northwest salmon‐bearing rivers, addressing concerns from resource‐limited managers tasked with salmon recovery. Using quantitative DNA metabarcoding of stomach contents and sampling across the Chinook salmon (*Oncorhynchus tshawytscha*) smolt outmigration phenology, we address three major questions: (1) what are the major predator–prey interactions for smallmouth bass, largemouth bass, rock bass and northern pikeminnow; (2) how does salmon availability (annual and seasonal) and water conditions influence the diet composition of each predator; and (3) do predator diets vary along the salmon outmigration corridor? These objectives are addressed across a spatially heterogeneous riverscape in the Chehalis River basin, Washington, USA, including both mainstem and tributary habitats, over multiple years that represent stark differences in discharge, stream temperature, and size of the Chinook salmon smolt outmigration. The Chehalis River is a focal point for salmon conservation and restoration, with significant investments aimed at recovering declining Chinook salmon populations. The Chehalis River is emblematic of the broader scale challenges facing freshwater ecosystems subject to growing populations of nonnative predators. We explore the implications of our findings for guiding Pacific salmon recovery efforts through targeted predatory fish control and highlight the importance of integrating multispecies perspectives into invasive species management.

## METHODS

### Study area

**FIGURE 1:**
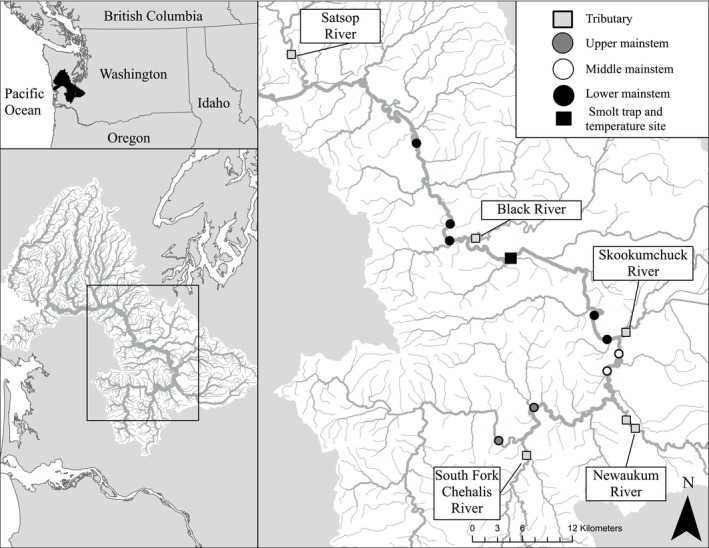
Map of Chehalis River and study reaches. Symbols represent sampling reaches and their relative locations (upper mainstem, middle mainstem, lower mainstem, and tributary) and smolt trap location where salmon smolt outmigration timing and abundance estimates were generated, and stream temperature data were collected.

The Chehalis River basin is a large coastal watershed in southwest Washington State, USA, that drains an approximately 7000‐km^2^ landscape into the Pacific Ocean through Grays Harbor (Figure 1). The basin's hydrologic regime is largely rain dominant, resulting in peak discharge in late fall and winter and protracted low discharge during summer months. Land use in the basin is generally private timberland in headwater locations (except for National Forest land in headwater sections of the Olympic Peninsula) and rural residential, agricultural, and urbanized in lowlands. The basin supports a diverse assemblage of freshwater species including Chinook salmon (fall and spring life history types), chum salmon (*Oncorhynchus keta*), coho salmon (*Oncorhynchus kisutch*), steelhead trout (*Oncorhynchus mykiss*), and coastal cutthroat trout (*Oncorhynchus clarkii*). While none of these fish populations are currently listed under the Endangered Species Act, petitions for federal protection of Chinook salmon on the west coast of the continental USA, inclusive of the Chehalis River, were recently submitted (NMFS, 2023). In the study area, Washington state‐managed hatchery supplementation programs are present for coho salmon and steelhead trout (Satsop River and Skookumchuck River) but not Chinook salmon.

### Interannual variation of Chinook salmon abundance and environmental conditions

Chinook salmon smolts outmigrate exclusively as subyearlings in the Chehalis River. Their outmigration exhibits a bimodal pattern, with an early fry outmigration from February to April, followed by a later parr group from late April through early July. The fry are believed to be less vulnerable to predation by centrarchids, as the bulk of their outmigration occurs before temperatures exceed known feeding thresholds for bass (e.g., <10°C; Stroud & Clepper, 1975). Therefore, we focus on the later migrating, and presumed more vulnerable, parr life stage (referred to as “smolt” from here forward).

Chinook salmon smolt population abundance is estimated annually from mark‐recapture data collected at a partial capture smolt trap in the mainstem Chehalis River (46.7982, −123.1643; Figure 1; West et al., 2020). Stream temperatures were monitored at the same location with a data logger (Onset HOBO Pendant 64K, Bourne, Massachusetts) recording at 30‐min intervals, providing important context for thermal conditions in the study area. Stream discharge data were collated from USGS stream gage 12027500 in the Chehalis River mainstem located centrally to the study area approximately 13 river kilometers upstream from the smolt trap (46.7751, −123.0353). We summarized these data into mean monthly values to describe inter‐ and intra‐annual variability in temperature and discharge (Table 1).

**TABLE 1:** Mean monthly stream temperatures (in degrees Celsius) and daily discharge (cubic meters per second) during the study period.

Month	Stream temperature (°C)		Discharge (m^3^/s)	
	2021	2022	2021	2022
May	15.8	12.3	18.6	80.1
June	20.2	15.7	18.2	57.4
July	22.3	22.2	6.2	16.6

*Note*: Stream temperature data were collected from a data logger recording at 30‐min intervals at 46.7982, −123.1643 and discharge data were summarized from USGS stream gage 12027500, located approximately 13 river kilometers upstream from the temperature monitoring location.

During the study, Chinook salmon smolt abundance was higher, stream temperatures were warmer, and stream discharge was lower in 2021 compared to 2022 (Table 1). Chinook salmon smolt abundance was estimated at 438,032 (CV = 7.0%) and 247,707 (CV = 7.5%) in 2021 and 2022, respectively. Mean monthly stream temperatures in the study area were 3.5°C warmer in May and 4.5°C warmer in June in 2021 compared to 2022 (Table 1). Discharge was, on average, three and a half times lower in 2021 compared to 2022 (Table 1). In both years, mean stream temperatures and discharge expectedly increased and decreased, respectively, as the Chinook salmon smolt outmigration progressed from early May through July.

### Data collection

#### Fish predator field sampling

**FIGURE 2:**
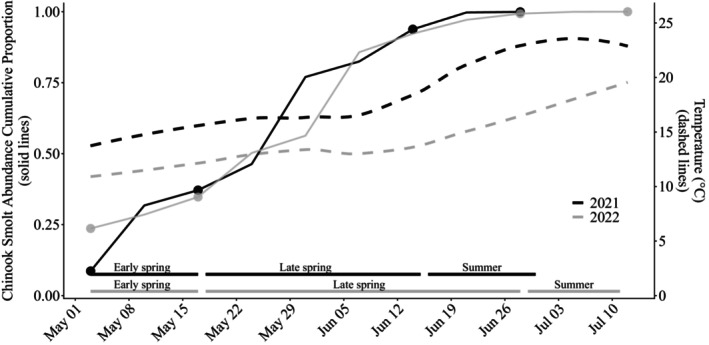
Chinook smolt cumulative abundance (proportion) (solid lines), mean weekly stream temperatures (in degrees Celsius) (dashed lines), and predator fish sampling periods (solid lines on the bottom of the figure) for 2021 and 2022. Circles on the solid lines correspond to the predator fish sampling periods.

Study reaches were embedded within salmon smolt migration corridors and the known centrarchid distributions of the basin (Winkowski et al., 2024; Zimmerman & Winkowski, 2022). However, reach selection was partially constrained by limited access suitable for launching and retrieving the sampling vessel. In total, 15 reaches were sampled during the spring and early summer of 2021 and 2022 and were located throughout upper, middle, and lower mainstem and tributary locations (Figures 1 and 2). Reach lengths were determined by multiplying the wetted width at the upstream end by 50, resulting in lengths ranging from 1.1 to 3.6 km.

To investigate intra‐annual predator diets across varying Chinook salmon smolt availability, we timed three sampling periods a priori to represent approximate early, peak, and late phases of the outmigration. These phases would presumably capture varying smolt availability (e.g., lower smolt abundance as the outmigration ascends, peak abundance, and lower smolt abundance as the outmigration descends). Based on historical smolt trapping data, Chinook salmon smolts begin migrating past the trap in early spring (late April to early May), peak in late spring (late May through mid‐ to late June), and finish by summer (early July). In 2021, the three sequential sampling periods captured approximately 9%–37%, 37%–94%, and 94%–99% of the cumulative proportional catch (Figure 2). In 2022, the corresponding coverage was 24%–35%, 37%–97%, and 97%–99% (Figure 2). Comparing our three sampling periods with the empirical outmigration data indicates that early, peak, and late phases were largely represented each year. We describe these seasonal sampling periods as early spring, late spring, and summer hereafter.

Dominant predatory fish species in the Chehalis River, also common to many other riverine systems in the Pacific Northwest, include nonnative smallmouth bass, largemouth bass, and rock bass and native northern pikeminnow. These species were the focus of the study and were collected by a raft‐mounted GPP 5.0 electrofisher (Smith‐Root, Vancouver WA, USA). All reaches were sampled by applying approximately one electrofishing “shot” per 100 m, with each shot defined as a 5‐min electrofishing effort (Kennard et al., 2011). Target species were collected, euthanized, and measured for fork length. To reduce cross‐contamination potential, separate sterile pipettes were used to inject 95% ethanol through the esophagus for DNA preservation of stomach contents (Brandl et al., 2016). Individual fish were placed in labeled bags on ice to slow digestive processes and preserve DNA until transferred to a freezer at the end of the sampling day (Brandl et al., 2016).

#### Fish predator stomach dissections

Dissections to remove stomach contents for DNA extraction were performed using aseptic technique intended to minimize the potential for sample contamination. Previously frozen fish were thawed overnight by placing bagged fish into a 1% bleach bath. Prior to each dissection, the workspace and all dissection tools were disinfected with a 10% bleach solution and thoroughly rinsed with deionized (DI) water. Stomachs were excised by making an incision on the ventral side of the fish from posterior to anterior, clamping either end of the stomach (esophagus and intestines) with hemostats, extracting the stomach with surgical scissors, and emptying the contents into labeled sterile Falcon conical tubes. Samples were immediately stored at −20°C until DNA extraction.

### DNA extraction, library preparation, sequencing, and bioinformatic analyses

All molecular work and bioinformatic analyses were performed by the Washington Department of Fish and Wildlife Molecular Genetics Laboratory (WDFW‐MGL, Olympia, WA, USA). Detailed methods for stomach content and mock community DNA extraction, library preparation, sequencing, and bioinformatic analyses can be found in Appendix S1: Section S1. To reduce the likelihood and quantify contamination during molecular work, each batch of individual stomach samples processed was paired with a negative control to detect any contamination between samples or of any extraction reagents. The extraction negative controls along with DNA template PCR negative controls were analyzed on every sample plate during library preparation and sequencing. Our results showed no fish DNA was present in any of the negative controls, confirming that stomach samples were free of cross‐contamination.

Briefly, DNA was extracted from lysed stomach contents and prey voucher specimens using a DNeasy Blood and Tissue Kit (QIAGEN). For DNA metabarcoding, we targeted a 313‐bp fragment of the mitochondrial cytochrome c oxidase subunit I (COI) gene region (Leray et al., 2013) for its ability to amplify numerous prey taxa that likely comprise the diets of our target predators including species of management and conservation interest and because COI is the most widely available sequence region in public reference libraries. Detailed library preparation methods can be found in Appendix S1: Section S1. Briefly, PCRs were performed in 30‐μL volumes using the Multiplex PCR Kit (QIAGEN) and included predator‐specific blocking oligonucleotides to limit amplification of predator DNA (Vestheim & Jarman, 2008). Amplicons were indexed, normalized, and pooled using the SequalPrep Normalization Plate Kit (Invitrogen). Amplicon libraries were cleaned, quantified, and normalized to 4 nM prior to sequencing on a MiSeq or NextSeq platform (Illumina).

Detailed bioinformatic methods can be found in Appendix S1: Section S1. Briefly, amplicon sequence data were demultiplexed, trimmed, and quality filtered in the QIIME 2 environment (Bolyen et al., 2019) using Cutadapt (Martin, 2011) and dada2 (Callahan et al., 2016). The resulting amplicon sequence variants (ASVs) were queried against a custom reference database, which included the MIDORI database (Machida et al., 2017), and assigned to the lowest possible operational taxonomic unit (OTU). Global taxonomic alignments between query and reference sequences were performed using the VSEARCH consensus taxonomy classifier (Rognes et al., 2016) and matches with ≥97% identity were retained.

We extracted, amplified, and sequenced DNA from 1065 stomach contents that contained prey DNA from smallmouth bass (mean = 254.0 mm, range = 80–455 mm, *n* = 150), largemouth bass (mean = 263.1 mm, range = 138–430 mm, *n* = 65), rock bass (mean = 144.4 mm, range = 55–240 mm, *n* = 431), and northern pikeminnow (mean = 272.2 mm, range = 75–530 mm, *n* = 419). An additional 23 analyzed samples contained no prey DNA (smallmouth bass = 2, largemouth bass = 0, rock bass = 7, northern pikeminnow = 14) likely due to complete digestion or degraded DNA and were not included in further analyses. The total number of samples collected varied for each predator by year, seasonal sampling period and location within years (Appendix S1: Table S1). On average across years and species, 6457 sequence reads were generated per sample. Species‐level assignments were achieved for 85.3% of the 481 OTUs.

To characterize prey items that were consistently consumed, we translated raw sequence reads of species‐level assignments to presence–absence and reported the frequency of occurrence of prey items detected in a minimum of 5% of the individuals. Then, we assessed if our sample sizes adequately characterized the most frequently occurring prey by developing rarefaction and sample completeness curves for each predator.

### Quantitative metabarcoding

Our goal was to move beyond presence–absence and compare the relative contribution of different prey to predator diets. However, bias introduced during the PCR process due to differential amplification efficiency and starting concentration of DNA template precludes such comparisons for metabarcoding data. To overcome this challenge, we calibrated our metabarcoding data with known mock communities of select prey DNA from vouchered specimens and Bayesian multinomial logistic regression models developed by Shelton et al. (2023). This modeling framework estimates species‐specific amplification efficiencies for selected prey based on the difference between the known starting and final proportions (post‐PCR and sequencing) of DNA in the mock community and applies these efficiencies to environmental samples to estimate true starting proportions of DNA (pre‐PCR). We constructed five mock communities with equal starting proportions of prey DNA that were sequenced in triplicate; four were constructed with a predator‐specific blocking oligonucleotide, consistent with the stomach content analysis, to account for potential PCR bias introduced by the blockers and one had no blockers to serve as a reference. Eleven fish species and three invertebrates were selected for the mock communities based on a combination of frequently occurring prey, species of socioeconomic and ecological significance, and availability of voucher tissues. Fish species included Chinook salmon, coho salmon, rainbow trout, prickly sculpin (*Cottus asper*), redside shiner (*Richardsonius balteatus*), Pacific lamprey, largescale sucker (*Catostomus macrocheilus*), and each of the four fish predators. Invertebrates included signal crayfish (*Pacifastacus leniusculus*) and representative species for mayfly from the genus *Diphetor*, and dragonfly from the genus *Ophiogomphus*. The benthic macroinvertebrate sequence counts, from the orders Ephemeroptera and Odonata, were pooled for each stomach content sample, and we used the representative species from the mock communities to estimate the pooled proportions. Amplification efficiencies likely vary among invertebrate species. However, collecting voucher specimens for all detected benthic macroinvertebrate species posed substantial logistical challenges, as they originated from 23 distinct families. Moreover, the number of species that can be included in a mock community is constrained by the compositional nature of the data, for example, adding too many species produces many rare taxa, which makes it difficult to determine whether zeros reflect sampling artifacts or poor amplification.

We ran the Shelton et al. (2023) model using three chains with 5000 iterations per chain and a tree length of 13. Posterior mean estimates of diet composition were reported with 95% CIs to compare dietary patterns among predators overall (averaged across years, seasons, and locations), interannually (averaged across seasons and locations), and intra‐annually by season and location. Aligned with our objectives, we focused primarily on salmon and other top contributing prey. All analyses and graphical procedures were conducted in R (R Development Core Team, 2024) with the packages tidyverse (Wickham et al., 2019), rstan (Guo et al., 2020), bipartite (Dormann et al., 2008), and iNEXT (Hsieh et al., 2016).

## RESULTS

Across all predator samples, we detected 39 ecologically relevant species. These included 11 fish species, signal crayfish, gastropods (*n* = 2), and 25 invertebrate species from the orders Trichoptera (*n* = 9), Diptera (*n* = 4), Ephemeroptera (*n* = 4), Haplotaxida (*n* = 3), Odonata (*n* = 2), Plecoptera (*n* = 1), Hemiptera (*n* = 1), and Hymenoptera (*n* = 1). We describe the diet contributions from gastropods and the 25 invertebrates (excluding signal crayfish) at the order level from here forward.

Prey frequency of occurrence varied among predators but also revealed notable dietary overlap and intraguild predation (Table 2, family level assignments in Appendix S1: Figure S1). Chinook salmon were frequently consumed by all predators (19%–41%), especially smallmouth bass (41% of samples). The smallest predators determined to consume Chinook salmon were 79 mm (rock bass), 80 mm (smallmouth bass; the smallest specimen collected), 100 mm (northern pikeminnow), and 150 mm (largemouth bass). Pacific lamprey were most frequently consumed by rock bass (15%) and Western brook lamprey (*Occidentis ayresii*) by smallmouth bass (5%, Table 2). Across predators, the most frequently consumed native fish prey were prickly sculpin (35%–71%) and largescale sucker (28%–40%; Table 2).

**TABLE 2:** Frequency of occurrence (proportion of samples, 0 = not detected in any sample, 1 = detected in all samples) for common prey in fish predator stomach contents.

Prey	Smallmouth bass	Largemouth bass	Rock bass	Northern pikeminnow
Native Fish				
Prickly sculpin	0.54	0.71	0.44	0.35
Largescale sucker	0.40	0.38	0.40	0.28
Chinook salmon	0.41	0.34	0.19	0.22
Pacific lamprey	0.05	0.09	0.15	0.08
Threespine stickleback	0.01	0.12	0.04	0.04
Torrent sculpin	0.06	0.05	0.05	0.04
Coho salmon	0.03	0.03	0.02	0.02
Intraguild				
Smallmouth bass	NA	0.88	0.67	0.62
Largemouth bass	0.41	NA	0.35	0.29
Rock bass	0.68	0.75	NA	0.57
Northern pikeminnow	0.44	0.46	0.34	NA
Crayfish				
Signal crayfish	0.51	0.62	0.62	0.59
Invertebrates				
Trichoptera	0.47	0.69	0.75	0.48
Ephemeroptera	0.16	0.14	0.48	0.18
Haplotaxida	0.14	0.12	0.22	0.12
Diptera	0.12	0.20	0.21	0.17
Gastropoda	0.18	0.22	0.20	0.15
Odonata	0.06	0.23	0.18	0.09
Hemiptera	0.04	0.05	0.06	0.03
Plecoptera	0.02	0.02	0.05	0.04
Hymenoptera	0.02	0.02	0.00	0.03

*Note*: Fish predators are in columns and prey are in rows. “NA” indicates no data are available for intraspecific predation.

Intraguild predation was prevalent, with predators frequently consuming each other (Table 2). Notably, all centrarchids were often consumed by native northern pikeminnow, as smallmouth bass, rock bass, and largemouth bass were detected in 62%, 57%, and 29% of diet samples, respectively. All predators frequently consumed invertebrate prey, with the highest rates observed for rock bass, specifically Trichoptera (75%), and Ephemeroptera (48%; Table 2).

Rarefaction curves reached asymptotes, and sample coverage was 100% for smallmouth bass, rock bass, and northern pikeminnow, indicating that our sample sizes adequately detected the 39 most frequently consumed prey (Appendix S1: Figure S2a). For largemouth bass, high sample coverage (98.2%) suggests that our sample size was nearly sufficient (Appendix S1: Figure S2b).

### Quantitative prey proportions in predator diets

For the mock communities, a few taxa closely matched their true abundance, while many were strongly over‐ or underrepresented (Appendix S1: Figure S3). These results demonstrate variable relative amplification efficiencies among the prey species, underscoring the importance of mock communities in calibrating DNA metabarcoding sequence reads to more accurately reflect true DNA composition in predator diets (Appendix S1: Figures S4–S7).

**FIGURE 3:**
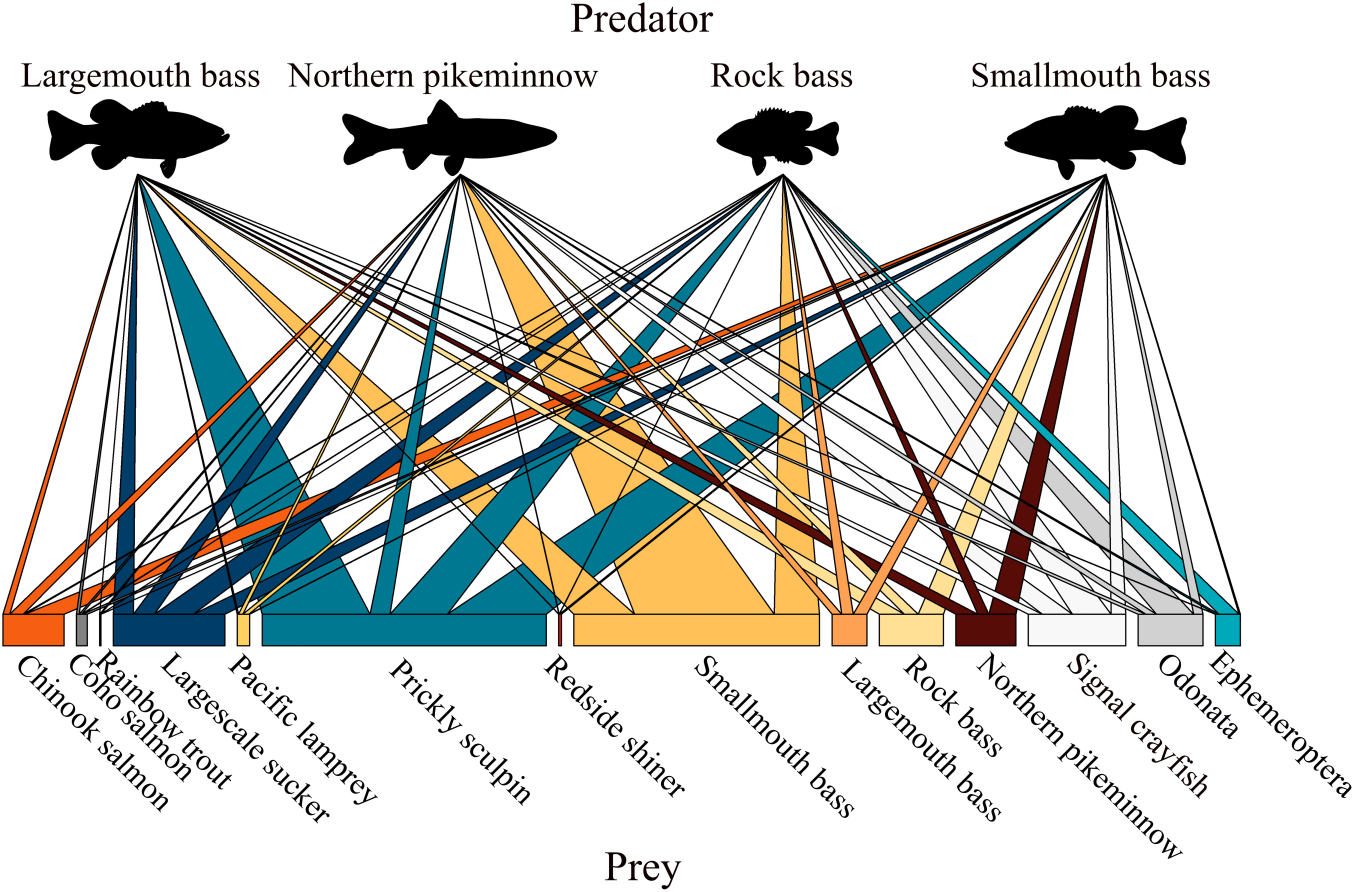
Bipartite network diagram displaying posterior mean estimates of overall prey contributions to fish predator diets. Predator–prey link sizes scale with the relative proportion of prey contributing to a predator's diet. Ephemeroptera and Odonata represent pooled proportions for all species in their respective order. Species silhouettes created by John J. Winkowski and Julian D. Olden.

Averaging across all samples, smallmouth bass diets were dominated by Chinook salmon and other native fishes (Figure 3). Prickly sculpin ranked highest (mean 36.8%, 95% CI 34.1%–42.5%), followed by Chinook salmon (mean 14.6%, 95% CI 13.9%–15.6%), largescale sucker (mean 11.8%, 95% CI 10.3%–13.9%), and northern pikeminnow (mean 10.0%, 95% CI, 7.9%–13.8%; Figure 3). Coho salmon contributed a smaller proportion relative to top prey items (mean 1.8%, 95% CI 1.6%–2.3%; Figure 3).

Largemouth bass diets were dominated by prickly sculpin (mean 40.1%, 95% CI 37.5%–43.1%), and smallmouth bass (mean 22.6%, 95% CI 20.2%–25.1%; Figure 3). Rock bass, northern pikeminnow, largescale sucker, and signal crayfish contributed similarly (5.2%–9.3%; Figure 3). Chinook (mean 2.5%, 95% CI 2.3%–2.7%) and coho salmon (mean 1.1%, 95% CI 1.0%–1.1%) contributed smaller proportions relative to top prey items (Figure 3).

For rock bass, prickly sculpin was the top prey contributor (mean 21.3%, 95% CI 19.6%–24.1%; Figure 3). Smallmouth bass, largescale sucker, signal crayfish, and Odonata all contributed relatively equally as the next most important prey (13.7%–16.9%; Figure 3). Chinook salmon contributions were relatively low compared to top prey items (mean 0.6%, 95% CI 0.5%–0.7%; Figure 3).

Northern pikeminnow diets were dominated by smallmouth bass (mean 51.9%, 95% CI 45.1%–63.9%; Figure 3). Their next highest prey was signal crayfish (mean 11.1%, 95% CI 8.4%–13.3%) followed by prickly sculpin (mean 7.4%, 95% CI 5.9%–9.9%), largescale sucker (mean 7.8%, 95% CI 5.3%–9.7%), and Chinook salmon (mean 5.0%, 95% CI 4.2%–6.2%; Figure 3). Coho salmon contributions were relatively low compared to other top prey (mean 0.9%, 95% CI 0.8%–0.9%; Figure 3).

### Interannual variability in predator diets

**FIGURE 4:**
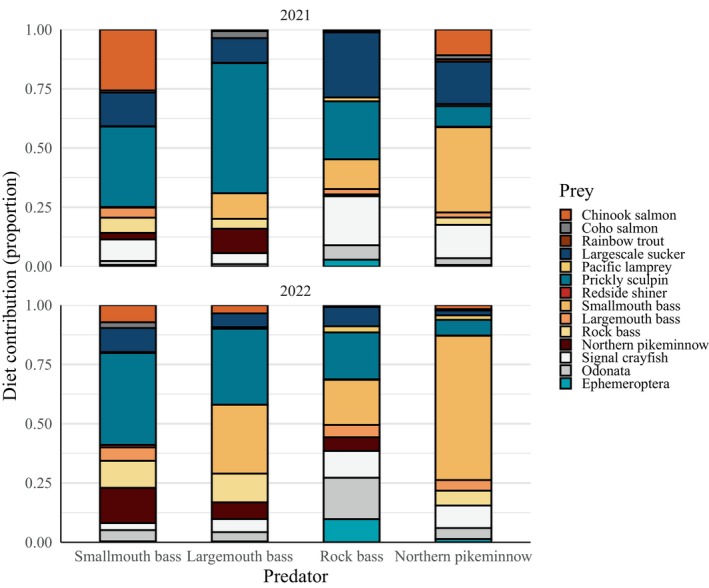
Posterior mean estimates of diet contributions by year. Ephemeroptera and Odonata represent pooled proportions for all species in their respective orders.

The overall top prey species for each predator were relatively consistent among years (Figure 4). However, notable variability was observed in the dietary contributions of Chinook salmon and intraguild predation. Consistent with being the top prey contributor to smallmouth bass diets overall, prickly sculpin was the dominant prey among years (mean range 33.9%–38.8%; Figure 4). In 2021, Chinook salmon were their second‐ranked prey (mean 25.7%, 95% CI 24.8%–27.0%), followed by largescale sucker (mean 14.2%, 95% CI 11.8%–17.3%; Figure 4). In 2022, their second highest prey contributor was northern pikeminnow (mean 14.8%, 95% CI 12.2%–18.4%), and Chinook salmon were their fifth‐ranked prey (mean 7.3%, 95% CI 6.6%–8.0%; Figure 4).

In 2021, top prey for largemouth bass were prickly sculpin (mean 54.9%, 95% CI 52.3%–57.7%) and smallmouth bass (mean 10.8%, 95% CI 8.7%–13.2%; Figure 4). Prickly sculpin and smallmouth were also the top two prey in 2022, but their contributions decreased and increased, respectively (Figure 4). Chinook salmon contributions were highest in 2022 (mean 3.5%, 95% CI 3.2%–3.7%; Figure 4).

For rock bass in 2021, largescale sucker, prickly sculpin, and signal crayfish were the top three prey contributors (mean 27.4%, 95% CI 25.3%–30.0%, mean 24.4%, 95% CI 22.6%–27.7%, mean 20.7%, 95% CI 18.8%–22.4%, respectively; Figure 4). In 2022, a broader assemblage of species comprised a similar proportion to the top three from 2021, including prickly sculpin (mean 19.8%, 95% CI 12.1%–22.4%), smallmouth bass (mean 19.0%, 95% CI 16.3%–21.9%), Odonata (mean 17.5%, 95% CI 15.8%–19.3%), signal crayfish (mean 11.3%, 95% CI 10.5%–12.2%), and Ephemeroptera (mean 9.7%, 95% CI 8.7%–10.9%; Figure 4). Chinook salmon contributions were low compared to top prey but were highest in 2021 (mean 0.9%, 95% CI 0.7%–1.0%; Figure 4). Pacific lamprey contributions were highest in 2022 (mean 2.6%, 95% CI 2.4%–2.9%; Figure 4).

Northern pikeminnow diets were dominated by smallmouth bass in both years but were higher in 2022 (mean 60.9%, 95% CI 55.4%–69.5%) compared to 2021 (mean 36.0%, 95% CI 26.9%–54.1%; Figure 4). Chinook salmon contributions were higher in 2021 (mean 10.9%, 95% CI 9.1%–13.1%) compared to 2022 (mean 1.7%, 95% CI 1.4%–2.4%; Figure 4).

### Intra‐annual variability in predator diets

**FIGURE 5:**
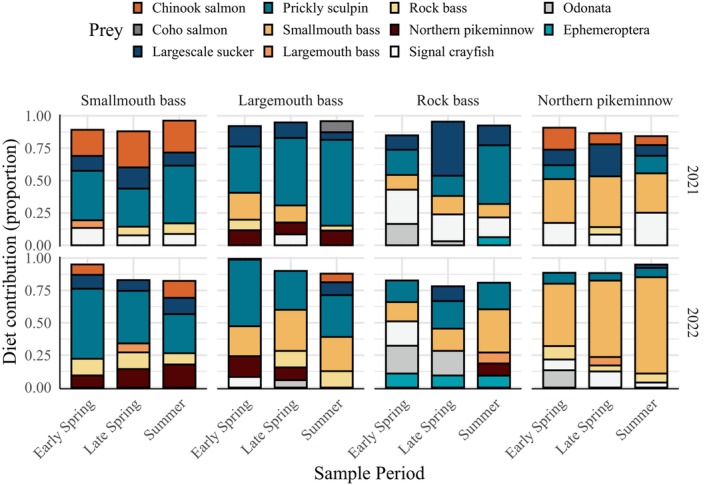
Posterior mean estimates of top five prey contributors per seasonal sampling period for fish predators in both years. Ephemeroptera and Odonata represent pooled proportions for all species in their respective order.

In general, predators consistently consumed their identified top overall prey through the progression of spring and summer, but some displayed dietary shifts (Figure 5). While Chinook salmon were hypothesized to drive seasonal diet shifts relative to their availability across their outmigration, limited evidence was detected.

Prickly sculpin were consistently the top prey for smallmouth bass throughout seasonal sampling periods both years (Figure 5). In 2021, Chinook salmon were the next most important prey across seasonal sampling periods and were only slightly elevated during their presumed peak outmigration in late spring (mean 27.9%, 95% CI 26.9%–29.3%; Figure 5). Largescale sucker contributions were also elevated in late spring of 2021 (mean 16.4%, 95% CI 13.8%–19.6%) compared to the other sampling periods (mean range 10.1%–11.5%; Figure 5). In 2022, the highest Chinook salmon contributions were during summer, when they were the third‐ranking prey for smallmouth bass (mean 13.1%, 95% CI 12.1%–14.1%; Figure 5).

Prickly sculpin were generally the top prey for largemouth bass throughout seasonal sampling periods both years (mean range 30.0%–66.3%; Figure 5). Their second and third most important prey were typically intraguild, including smallmouth bass and northern pikeminnow. Largescale sucker were also important prey across seasons, specifically in 2021 (Figure 5). The highest Chinook salmon contributions were during the summer of 2022, when they were the fifth‐ranked prey item (mean 6.7%, 95% CI 6.4%–7.0%; Figure 5).

Rock bass displayed the most distinct shifts of top‐ranking prey both years. In 2021, the top‐ranking prey shifted from signal crayfish in early spring (mean 26.5%, 95% CI 23.8%–29.4%) to largescale sucker in late spring (mean 41.7%, 95% CI 39.3%–44.6%) to prickly sculpin in summer (mean 45.3%, 95% CI 43.6%–47.8%; Figure 5). In 2022, their most important prey shifted from Odonata in early spring (21.5%, 95% CI 19.1%–23.9%) to prickly sculpin (21.3%, 95% CI 19.7%–23.6%) and Odonata (19.0%, 95% CI 17.4%–20.8%) in late spring to smallmouth bass (33.4%, 95% CI 29.0%–37.6%) and prickly sculpin (20.5%, 95% CI 18.3%–23.9%) in summer (Figure 5).

For northern pikeminnow, smallmouth bass were consistently the highest prey contributor across seasonal sampling periods both years (mean range 30.4%–77.4%; Figure 5). In 2021, Chinook salmon were the second‐ranked prey in early spring (mean 17.0%, 95% CI 15.1%–19.7%), but this shifted to largescale sucker in late spring (mean 24.8%, 95% CI 17.3%–30.2%), and signal crayfish in summer (mean 25.2%, 95% CI 20.1%–29.6%), as Chinook diet contributions progressively declined (Figure 5). In 2022, prickly sculpin and signal crayfish were consistently the second or third highest contributors (mean range 3.8%–12.4%) across sampling periods (Figure 5).

**FIGURE 6:**
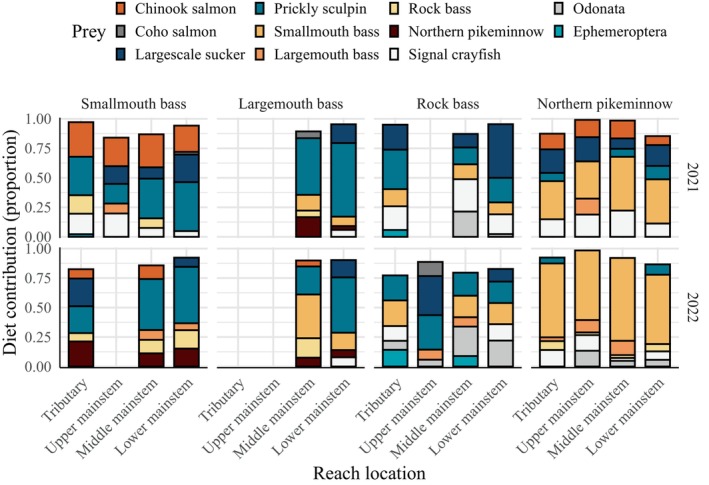
Posterior mean estimates of top five prey contributors per reach location for fish predators both years. Missing bars indicate ≤1 predator fish sample collected. Ephemeroptera and Odonata represent pooled proportions for all species in their respective order.

Diet compositions across reach locations were generally consistent with predators' top overall prey both years (Figure 6). However, some notable spatiotemporal patterns emerged related to Chinook salmon consumption by smallmouth bass. For smallmouth bass in 2021, while Chinook salmon diet contributions were relatively consistent across reach locations, elevated dietary contributions were apparent in the middle mainstem (mean 28.0%, 95% CI 27.1%–29.6%) and tributary reaches (mean 29.3%, 95% CI 28.4%–30.8%) compared to the upper (mean 24.2%, 95% CI 22.9%–25.6%) and lower mainstem reaches (mean 22.3%, 95% CI 21.6%–23.2%; Figure 6). In 2022, consumption of Chinook salmon decreased across reach locations but remained highest in the middle mainstem (mean 11.6%, 95% CI 10.6%–12.6%) and tributary reaches (7.9%, 95% CI 7.3%–8.5%; Figure 6). Consistent with their top‐ranking prey overall, prickly sculpin were among the top contributors to smallmouth bass diets for all reach locations both years (Figure 6).

Largemouth bass were only captured in middle and lower mainstem reaches both years. Consistent with their dominant prey overall, prickly sculpin were generally the largest prey contributor across locations. Diet contributions of Chinook salmon were generally low but highest in middle mainstem reaches in 2022 (mean 5.1%, 95% CI 4.8%–5.4%; Figure 6).

For rock bass, dominant prey varied among reach locations. Prickly sculpin, largescale sucker, Odonata, signal crayfish, and smallmouth bass were all top contributors (Figure 6). The highest contributions of Chinook salmon were in the lower (1.5%, 95% CI 1.4%–1.8%) and middle mainstem (1.1%, 95% CI 0.7%–1.5%) in 2021.

For northern pikeminnow, smallmouth bass were the top prey contributor across reach locations both years (Figure 6). In 2021, Chinook salmon consumption by northern pikeminnow was relatively consistent across upper and middle mainstem and tributary reaches (13.3%–15.2%) and lowest in lower mainstem reaches (mean 7.6%, 95% CI 6.0%–9.6%; Figure 6). In 2022, Chinook salmon contributions were relatively low across all reach locations (≤2.3%).

## DISCUSSION

Our findings underscore the known threat of smallmouth bass to Pacific salmon conservation and recovery, while also highlighting broader ecological impacts from a growing number of other nonnative gamefish (Carey et al., 2011; Murphy et al., 2021; Sanderson et al., 2009). Collectively, Chinook salmon were frequently consumed by all predators, with the highest relative proportions in smallmouth bass diets. Moreover, smallmouth bass consumed Chinook salmon more frequently and at smaller body sizes than those typically considered in previous regional diet analyses (Fritts & Pearsons, 2004; Tabor et al., 2007; Zimmerman, 1999). While nonnative largemouth and smallmouth bass are known primary predators on Chinook salmon in the Pacific Northwest, our study provides novel information on predation risk from nonnative rock bass. Though mostly overlooked in regional predation assessments, rock bass are effective opportunistic predators, feeding at both day and night (Keast & Welsh, 1968), and capable of consuming large prey relative to their size (Keast & Webb, 1966). Although contributing in lower relative proportions, Chinook salmon were a common diet item for rock bass and were consumed by relatively small individuals. While broader population assessments across the Pacific Northwest are unavailable, rock bass are the most abundant centrarchid in the Chehalis River, meaning even relatively low levels of predation are alarming (WDFW, unpublished data).

Other salmon species were less frequently consumed and accounted for smaller proportions of diets compared to other top prey during the seasonal period captured by our study, though some predation still occurred. Compared to Chinook salmon smolts, Coho salmon and steelhead trout smolts are less abundant and generally migrate after 1–3 years of rearing, reaching substantially larger sizes (West et al., 2020). These smolts can likely evade predators more effectively and may exceed gape limitations of some of the smaller‐sized fish predators. During spring and summer, smaller‐sized non‐migrating coho salmon and steelhead trout parr face less predation risk by nonnative fishes because of minimal spatial overlap (Winkowski et al., 2024; Winkowski & Zimmerman, 2018; Zimmerman & Winkowski, 2022). Downstream autumn migrations of coho salmon and steelhead trout parr may provide an asynchronous and complementary prey pulse to the spring and summer Chinook salmon smolt outmigration. This could enable nonnative predators to exploit salmon prey across seasonal feeding periods, a perspective largely unexamined across rivers of the Pacific Northwest.

Chinook salmon contributions to predator diets were generally higher during a year of lower discharge and warmer temperatures, potentially foreshadowing intensified predation pressures as climate change enhances conditions for nonnative predators (Jan et al., 2025). Warmer stream temperatures, like those experienced in 2021, increase activity, metabolism, and consumption rates of warmwater fish predators (Lawrence et al., 2015; Vigg & Burley, 1991) while simultaneously hindering physiological processes of Chinook salmon (Kuehne et al., 2012). Moreover, low discharge reduces turbidity and improves predator foraging success by increasing prey accessibility and encounter rates, as documented for predators who rely on vision to locate and capture prey like smallmouth bass (Sweka & Hartman, 2003) and other piscivorous fish more broadly (Abrahams & Kattenfeld, 1997). By contrast, the cooler and higher discharge conditions in 2022 likely presented less favorable foraging conditions and may help to explain the relatively low contributions of Chinook salmon to predator diets. In 2022, consumption of Chinook salmon by largemouth and smallmouth bass peaked during the summer sampling period, concurrent with the warmest stream temperatures and lowest discharge. Since environmental conditions and Chinook salmon abundance varied among years, it is difficult to untangle their individual effects on dietary patterns. However, these observations highlight how environmental conditions can shape predation pressures by nonnative fish predators, emphasizing the urgency of addressing this issue for Chinook salmon as climate change intensifies both regionally (Winkowski, 2023) and more broadly across their range (Isaak et al., 2017).

Predation risks for migratory prey vary as they navigate spatially diverse landscapes (Sabal et al., 2021). Adopting a spatial perspective is essential for understanding these risks across heterogeneous riverscapes and can guide the prioritization of resources aimed at reducing predation pressure on salmon (Michel et al., 2020). Across years of varying salmon availability and environmental conditions, we identified consistently high contributions of Chinook salmon to predator diets in the middle mainstem and tributaries, suggesting these reaches may represent predation hotspots. The sampled tributaries are critical habitat for declining spring Chinook salmon in the Chehalis River, representing nearly 80% of the spawning population over the last 20 years (WDFW, unpublished data). The middle mainstem region is geomorphologically unique, characterized by low‐gradient, homogeneous, and slow‐moving habitats. These more lentic‐like river reaches may have elevated predation risk compared to more complex lotic river reaches (Buchanan et al., 1981), in part due to increased spatial (e.g., shoreline habitats) and temporal (e.g., slower salmon smolt outmigration times) overlap between Chinook salmon smolts and warmwater predators, as well as generally favorable foraging conditions (Tiffan et al., 2016). Moreover, this region hosts some of the highest densities of smallmouth bass throughout their distribution (WDFW, unpublished data), likely magnifying predation pressure through this mainstem corridor.

Phenological synchrony, combined with invaders' biological characteristics and climate matching between regions, is a key factor facilitating invasive predator successes in new ecosystems (Fausch et al., 2001; Moyle & Marchetti, 2006). During spring, synchronous prey phenologies, including salmon smolt migrations and native fish reproduction (e.g., availability of eggs and newly hatched fry), provide timely prey pulses that likely benefit nonnative predators as they ramp up feeding after winter to meet reproductive energy demands (Fritts & Pearsons, 2008). Our study provides evidence that Chinook salmon may benefit from a prey buffering effect provided by abundant native species. Generalist predators typically target abundant prey, and prickly sculpin and largescale suckers offer synchronous prey sources during the salmon smolt migration. In our study, these species were consistently top‐ranking diet items throughout the Chinook salmon smolt outmigration and across reach locations. Similar buffer mechanisms have been postulated in the Columbia River, where abundant sand rollers (*Percopsis transmontana*), crayfish, and other small invertebrates may reduce smallmouth bass predation pressure on Chinook salmon smolts (Hemingway et al., 2019; Tiffan et al., 2020). Buffer mechanisms are increasingly recognized as pivotal ecosystem management strategies (Milles et al., 2023). Our findings support the idea that native species populations may indirectly influence Chinook salmon survival and emphasize the need for monitoring and conservation efforts focused on often‐overlooked native species.

Our study highlights the taxonomic diversity of prey consumed by nonnative fish, underscoring their broad influence and complex roles within freshwater food webs (Eby et al., 2006; Naiman et al., 2012). Part of this complexity stems from indirect predation impacts. For example, we demonstrate that prickly sculpin constitutes a significant component of nonnative predator diets. Cottids figure prominently in Pacific Northwest freshwater ecosystems due to their wide distribution, abundance, value as prey, and role as opportunistic predators of macroinvertebrates, small fish, and eggs. They also serve as critical hosts for the parasitic larval stage of imperiled native freshwater mussels (Maine & O'Brien, 2022). Despite these critical ecosystem functions, little is known about the status of cottid populations across their range in the region. While the overall impact of nonnative predators on cottids has not been quantified, our findings suggest predation pressure may be substantial, with potential indirect effects on ecosystem structure and function. Similarly, aquatic gastropods and signal crayfish were frequently consumed by all nonnative predators, indicating predation pressures on lower trophic organisms whose suppression can trigger cascading ecological impacts (Jackson et al., 2014).

Our results shed new light on predator interactions with implications for management. First, our study illustrates the threat of nonnative fish to other species of conservation concern. Native lamprey (family *Petromyzontidae*) are declining in the Pacific Northwest, sparking petitions for conserving these culturally and ecologically vital species (Clemens et al., 2017). Among multiple stressors threatening populations, our study supports recent evidence identifying nonnative fish predation as a key factor (Arakawa & Lampman, 2020; Schultz et al., 2017). Second, our study detected strong intraguild predation interactions in the food web, differing from previous fish predation studies in the Pacific Northwest (Poe et al., 1991; Rieman et al., 1991; Zimmerman, 1999). One possible explanation is intraguild predation on early life stages, which is challenging to identify using visual methods relied upon for historical studies (Schooley et al., 2008). For example, northern pikeminnow and centrarchids have been documented to consume fish eggs and fry (Beauchamp et al., 1995; Tabor et al., 2007) and in their native range, recruitment failures of smallmouth bass have been linked to piscine nest predation (Dauwalter & Fisher, 2007). Moreover, historical dietary studies in the region generally focused on larger adult fish predators, with less emphasis on smaller individuals that may prey disproportionately on fish eggs and early life stages (Zimmerman, 1999).

Strong intraguild predation links support the hypothesis that northern pikeminnow may play, at least some role, in regulating nonnative fish predators. Stable isotope analysis similarly suggests that northern pikeminnow derive substantial dietary input from nonnative gamefish in the Columbia River (Murphy et al., 2021). Although smallmouth bass densities varied spatially, they were consistently northern pikeminnow's dominant prey, potentially suggesting targeted predation. The Northern Pikeminnow Management Program has removed northern pikeminnow for over 30 years in the Columbia River, based on earlier evidence indicating they were the primary salmon predator, but with limited knowledge of intraguild interactions. In areas where northern pikeminnow have been significantly reduced, recent monitoring trends suggest potential compensatory responses from nonnative predator populations, including increased abundance and salmon predation by smallmouth bass (Winther et al., 2023). If reducing one predator allows nontarget predators to maintain or increase predation on imperiled salmon, the goals of such programs may be compromised. Our findings illustrate how single‐species predator control may be undermined by interactions within the predator guild and demonstrate the importance of characterizing these dynamics (Bergstrom et al., 2009; Lurgi et al., 2018; Zavaleta et al., 2001).

In our study, dietary DNA metabarcoding was effective for describing complex predator–prey links in novel food web assemblages. However, this approach has limitations that are shared with other approaches, such as stable isotope analyses. DNA metabarcoding cannot determine prey size or life stage, limiting detailed insights into predator–prey interactions. Another concern is secondary predation, where DNA from prey of ingested animals can lead to false positives (Sheppard & Harwood, 2005). Our molecular approach cannot distinguish cannibalism, thereby limiting our understanding of predator diets (Cuff et al., 2023). Additionally, while accounting for amplification bias helps bridge the gap between translating metabarcoding data to quantitative impacts, incorporating factors that affect DNA degradation through digestion remains essential, including DNA detectability half‐lives, species‐specific digestion rates, and environmental variables like temperature (Brandl et al., 2016; Dick et al., 2023). Recent advances in statistical modeling offer promising frameworks, accounting for both species‐specific amplification efficiencies and conversion parameters related to environmental DNA persistence (Guri et al., 2024).

Our study investigated the impacts of a common assemblage of co‐occurring nonnative and native fish predators in Pacific Northwest rivers. Building on these findings, contemporary information is needed on other expanding but understudied nonnative fish in the region, such as yellow perch (*Perca flavescens*) and walleye (*Sander vitreus*). Salmon smolts also face increasing predation threats from avian and mammalian predators, especially around dams, further intensifying the predation landscape during their freshwater life stages and adding complexity to the food web. Additionally, natural resource managers in the Pacific Northwest face the challenging paradox of protecting culturally, ecologically, and socioeconomically important species like salmon while supporting popular and lucrative recreational angling for nonnative gamefish. Addressing this challenge may be aided by understanding how novel predator assemblages function to promote the restoration of food webs that support native species while minimizing the likelihood of compensatory effects. As ecosystems worldwide face increasing pressures from co‐occurring invasive species, integrating multispecies approaches into management strategies is essential for mitigating impacts and conserving biodiversity. These approaches will not only aid in conservation efforts for Pacific salmon but also support the resilience of other ecosystems challenged by co‐occurring invasive species.

## CONFLICT OF INTEREST STATEMENT

The authors declare no conflicts of interest.

## Supporting information


Appendix S1.

## Data Availability

Data and code (Winkowski et al., 2025) are available in Figshare at https://doi.org/10.6084/m9.figshare.28498745.v1.
