# Supplementary material for: Unraveling multipredator impacts in salmon‐bearing rivers using quantitative DNA metabarcoding
Source: Ecol Appl. 2025 Dec 18;35(8):e70158. doi: 10.1002/eap.70158 (PMC12715301; doi:10.1002/eap.70158)
Supplement: Supplementary file 1 — Appendix S1. [file EAP-35-e70158-s001.pdf]

Authors: John J. Winkowski, Lisa M. Crosson, Julian D. Olden

Title: Unraveling multipredator impacts in salmon-bearing rivers using quantitative DNA metabarcoding

Journal: Ecological Applications

Appendix S1

Section S1 – DNA extraction, library preparation, sequencing, and bioinformatic analyses

DNA Extraction - stomach contents

All DNA extractions were conducted in AirClean® 600 PCR Workstations equipped with HEPA air filtration and UV-C light to minimize sample contamination. Prior to extraction, all surfaces and equipment were disinfected with a 10% bleach solution and exposed to UV-C light for one hour to neutralize exogenous DNA. Stomach contents were lysed for 48hrs at 56°C with periodic vortexing in 4.5mL of Buffer ATL (QIAGEN) and 0.5mL of proteinase K. DNA was extracted from the lysate using a DNeasy® Blood & Tissue Kit (QIAGEN) according to the manufacturers protocol for purification of total DNA from animal tissues and eluted in 100µL final volume. One extraction negative control was processed per batch of 23 samples and all DNA was stored at -20°C prior to library preparation.

Mock Community Construction

Preserved tissues from select prey voucher specimens were aseptically sampled and DNA extracted using a DNeasy® Blood & Tissue Kit (QIAGEN) according to the manufacturers protocol for purification of total DNA from animal tissues and eluted in 100µL final volume. Genomic DNA was normalized to a concentration of 10ng/µL using a Qubit® fluorometer (Life

Technologies) and five mock communities comprising 11 fish species and 3 invertebrates (Chinook salmon (*Oncorhynchus tshawytscha*), coho salmon (*O. kisutch*), rainbow trout (*O. mykiss*), prickly sculpin (*Cottus asper*), redside shiner (*Richardsonius balteatus*), Pacific lamprey (*Entosphenus tridentatus*), largescale sucker (*Catostomus macrocheilus*), smallmouth bass (*Micropterus dolomieu*), largemouth bass (*M. salmoides*), rock bass *(Ambloplites rupestris*), northern pikeminnow (*Ptychocheilus oregonensis*), signal crayfish (*Pacifastacus leniusculus*) and representative species for mayfly from the genus *Diphetor*, and dragonfly from the genus *Ophiogomphus*) were constructed with equal proportions of prey DNA. Four communities were constructed with a blocking oligonucleotide for each predator species (largemouth bass, smallmouth bass, rock bass, and northern pikeminnow) and one community with no blocker to serve as a reference. All five mock communities were sequenced in triplicate.

Library Preparation and Sequencing

A 313bp fragment of the mitochondrial cytochrome c oxidase subunit I (COI) gene region was amplified using primers developed by Leray et al. (2013)and included the forward primer mlCO1intF 5′-GGWACWGGWTGAACWGTWTAYCCYCC-3′ and the reverse primer jgHCO2198 5′-TAIACYTCIGGRTGICCRAARAAYCA-3′ (Geller et al. 2013).

PCRs were performed in 30µL volumes using the Multiplex PCR Kit (QIAGEN). Reactions contained 15µL (1X) of multiplex master mix, 0.8µM of each primer, 1µM of predator specific blocking oligonucleotide, and 2µL of template DNA with thermal cycling conditions as follows: 95°C for 10min, 35 cycles of 95°C for 60s, 50°C for 60s, and 72°C for 60s, and a final extension of 72°C for 5min. A PCR negative template control (NTC) and positive control were included on

each 96-well PCR plate. The NTC consisted of sterile molecular grade water in lieu of template DNA and kangaroo DNA was used as a positive amplification control.

PCR products were size selected using Mag-Bind® TotalPure NGS (Omega Biotek) beads at a 0.8X ratio of beads to product.. Sample amplicons were then indexed with Nextera DNA unique dual (UD) indexes (IDT® for Illumina®), normalized using the SequalPrep™ Normalization Plate Kit (Invitrogen), and pooled. Plate libraries were bead cleaned at a 0.8X bead ratio, quantified with a Qubit® fluorometer (Life Technologies), and normalized to 4nM prior to loading. Stomach content libraries were sequenced on the MiSeq™ platform using the MiSeq™ v2 (300 cycle) Reagent Kit for single end reads with a 5% PhiX Control v3 Library (Illumina) and mock community libraries were sequenced on the NextSeq™ 1000 platform using the NextSeq™ 1000 P1 (600 cycles) Reagent Kit for paired end reads with 25% PhiX Control v3 Library (Illumina).

Bioinformatic Analysis

Amplicon sequence data was analyzed using either stand-alone QIIME 2 (Bolyen et al. 2019) and DADA2 (Callahan et al. 2016) or with Tourmaline (https://github.com/aomlomics/tourmaline), a Snakemake pipeline that wraps QIIME 2 and DADA2, providing reproducible metabarcoding analysis. Adapters and primers were trimmed from demultiplexed FASTQ reads using Cutadapt (Martin 2011). The program DADA2 was used to quality filter reads. Reads were truncated to a common length of 200bp with a maximum number of expected errors = 2, chimeras were removed using the consensus method, and amplicon sequence variants (ASVs) were exported. To assign COI taxonomy, a custom reference database was generated to include the MIDORI database (Machida et al. 2017), containing over 580,000 nucleotide sequences, and all mitochondrial COI sequences in the National Center for Biotechnology Information (NCBI) nucleotide database. Global taxonomic alignments between

query and reference sequences were performed using the VSEARCH consensus taxonomy classifier (Rognes et al. 2016) and matches with ≥97% identity were retained.

Table S1. Number samples analyzed for diet composition for each predator by year ("total analyzed" row) and seasonal sampling periods and locations within years.

	Smallmouth bass		Largemouth bass		Rock bass		Northern pikeminnow	
	2021	2022	2021	2022	2021	2022	2021	2022
Total analyzed	61	89	23	42	145	286	153	266
Seasonal sampling								
Early spring	15	12	3	2	34	86	47	49
Late spring	36	46	12	22	70	156	80	150
Summer	10	31	8	18	41	44	26	67
Sampling location								
Tributary	5	23	0	0	60	100	60	91
Upper mainstem	8	1	0	0	0	6	7	12
Middle mainstem	27	39	12	27	33	68	14	19
Lower mainstem	21	26	11	15	52	112	72	144

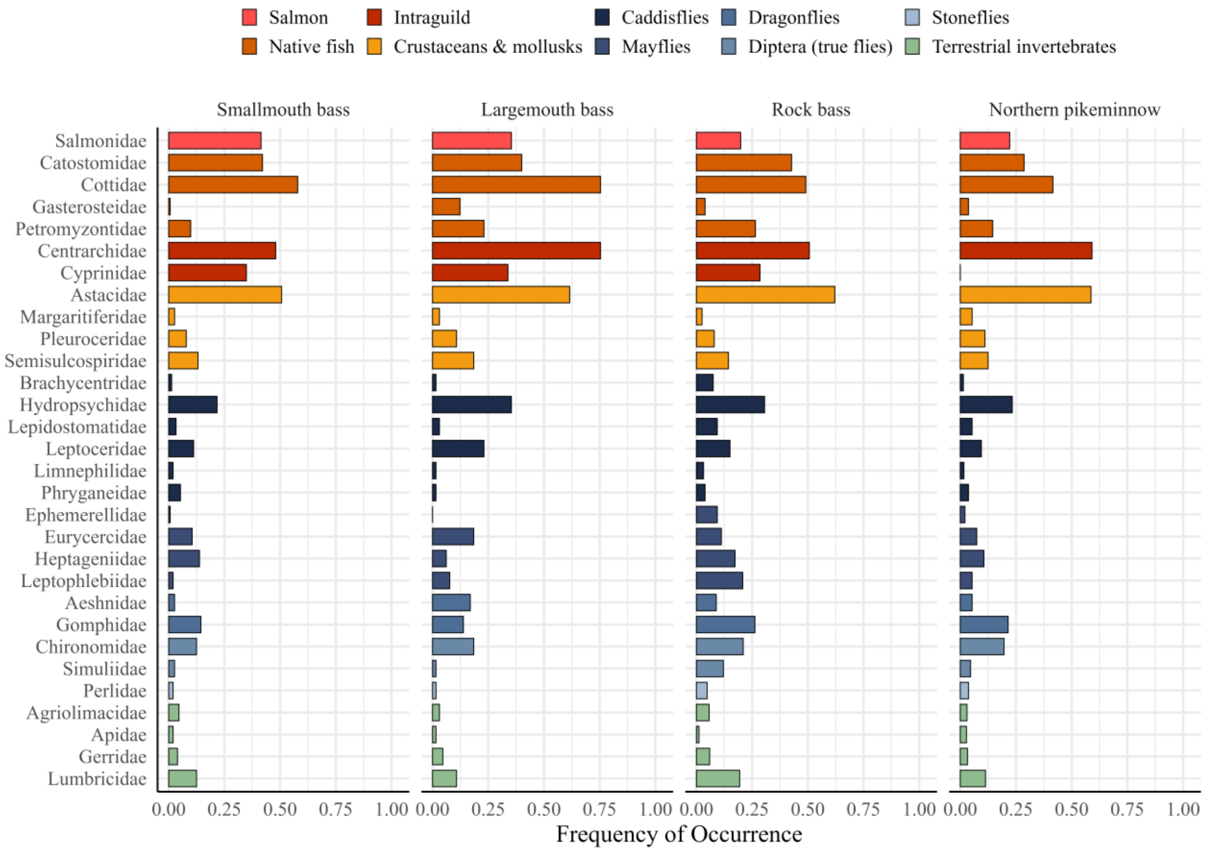

Figure S1. Frequency of occurrence of common prey at the family level for each predator.

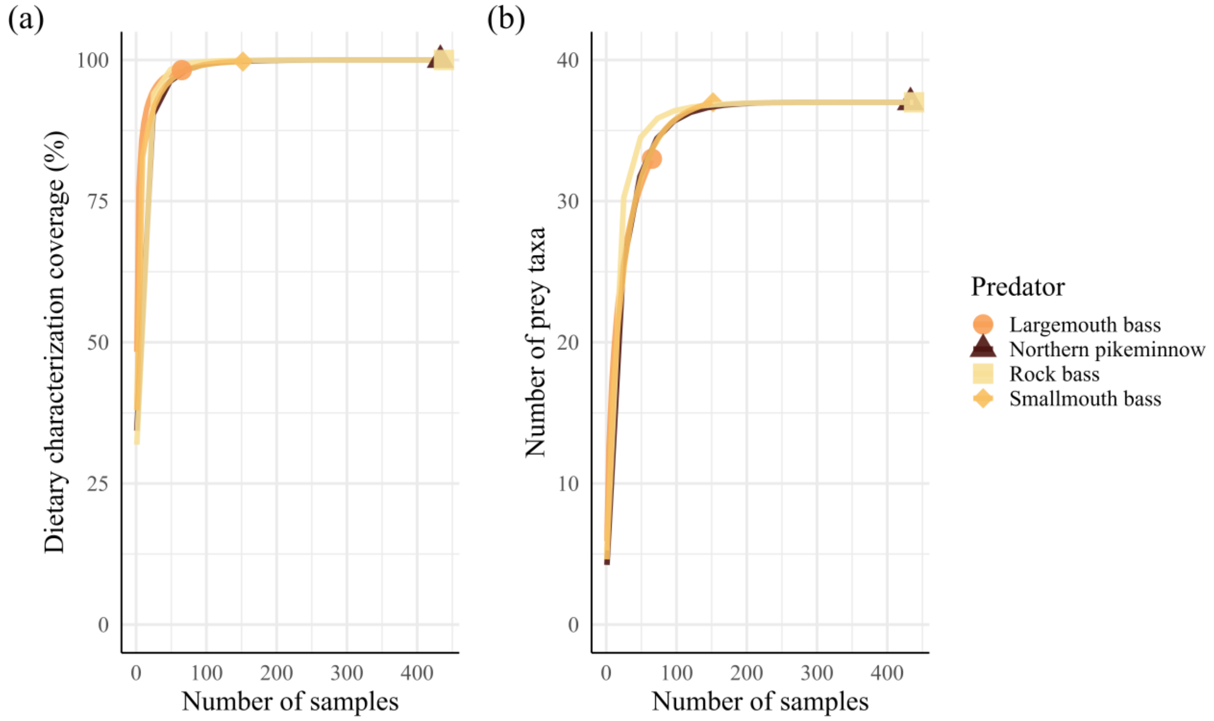

Figure S2. a) Rarefaction and b) sample completeness curves for each predator.

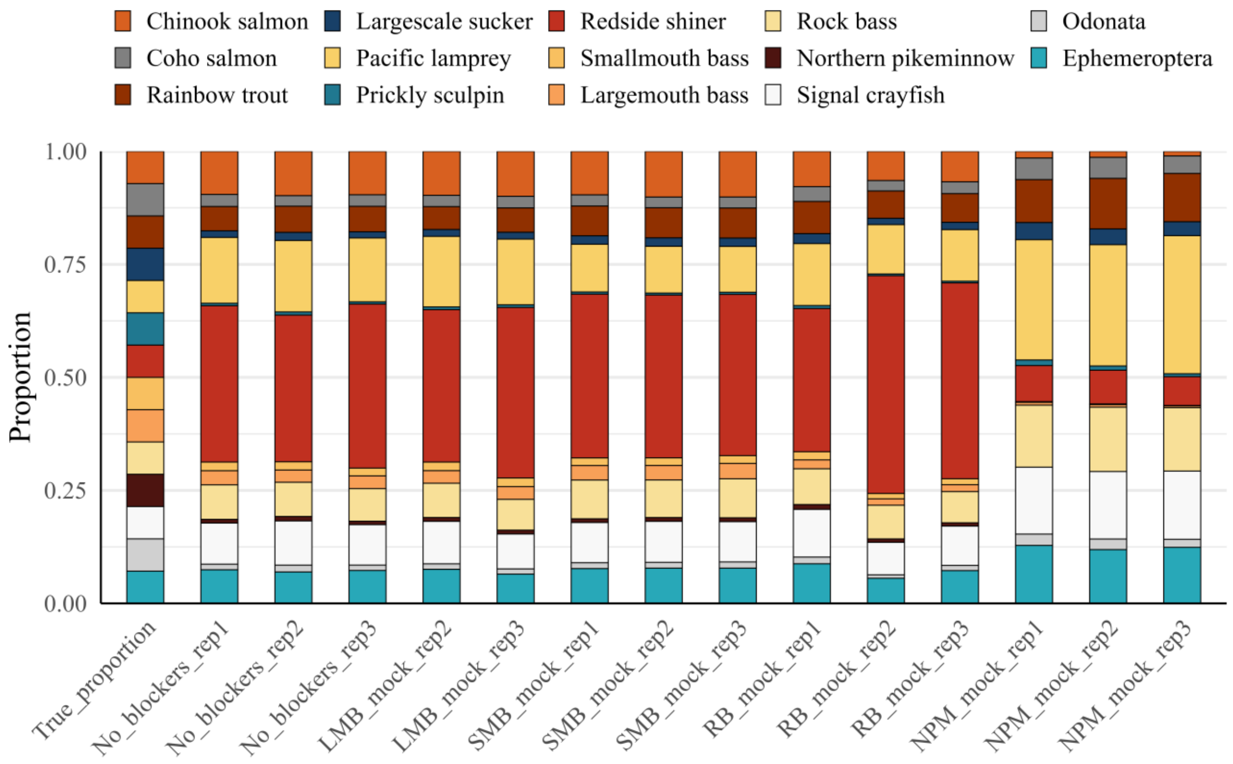

Figure S3. DNA composition (proportion) of starting mock community ("True_proportion") and mock communities with and without predator specific blockers after amplification and sequencing (LMB = largemouth bass, SMB = smallmouth bass, RB = rock bass, and NPM = northern pikeminnow).

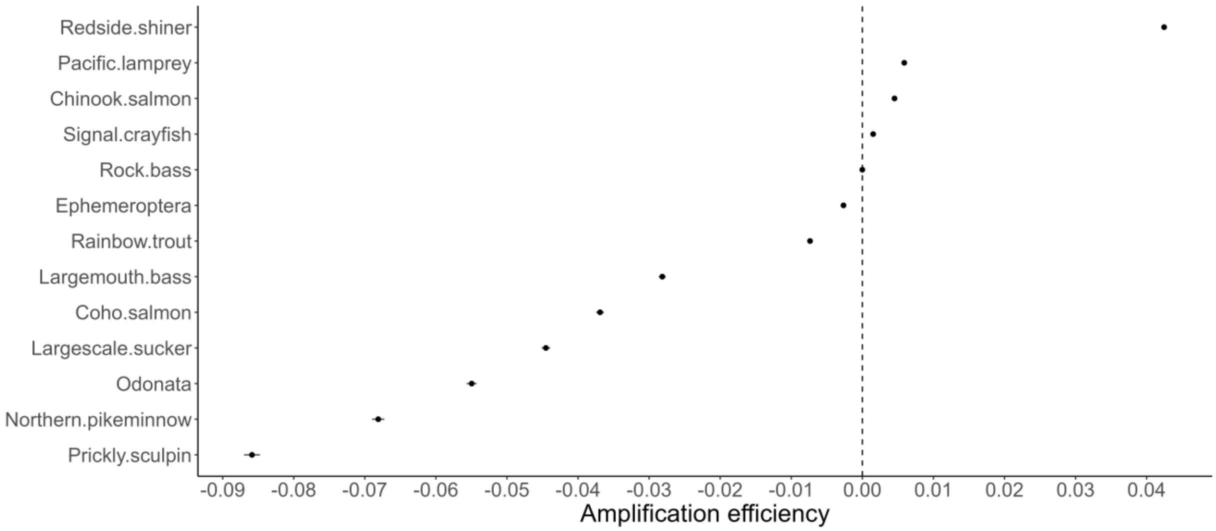

Figure S4. Smallmouth bass mock community posterior amplification efficiency point estimates and 95% confidence intervals.

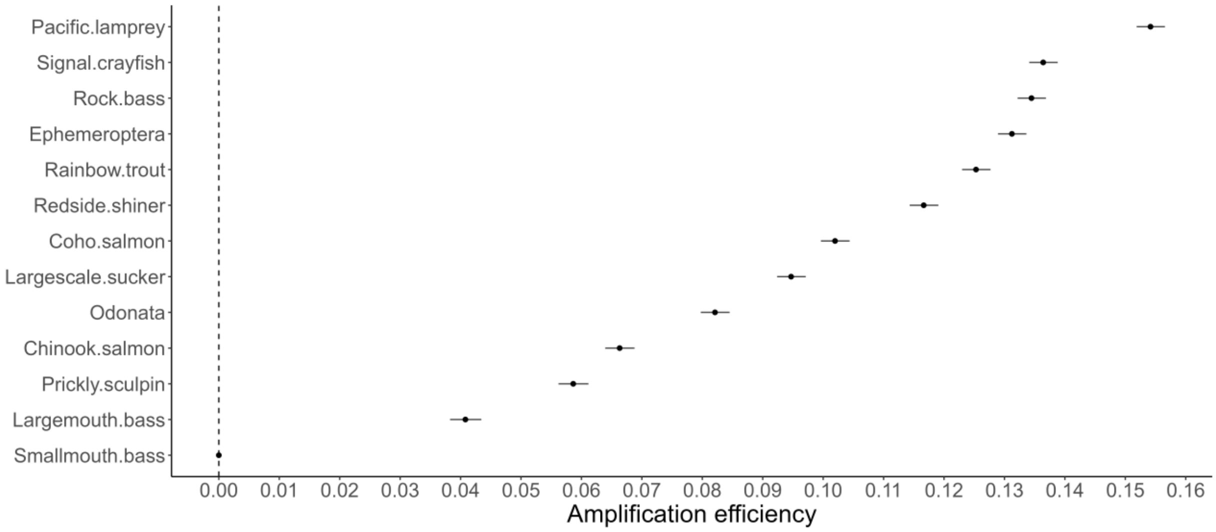

Figure S5. Northern pikeminnow mock community posterior amplification efficiency point estimates and 95% confidence intervals.

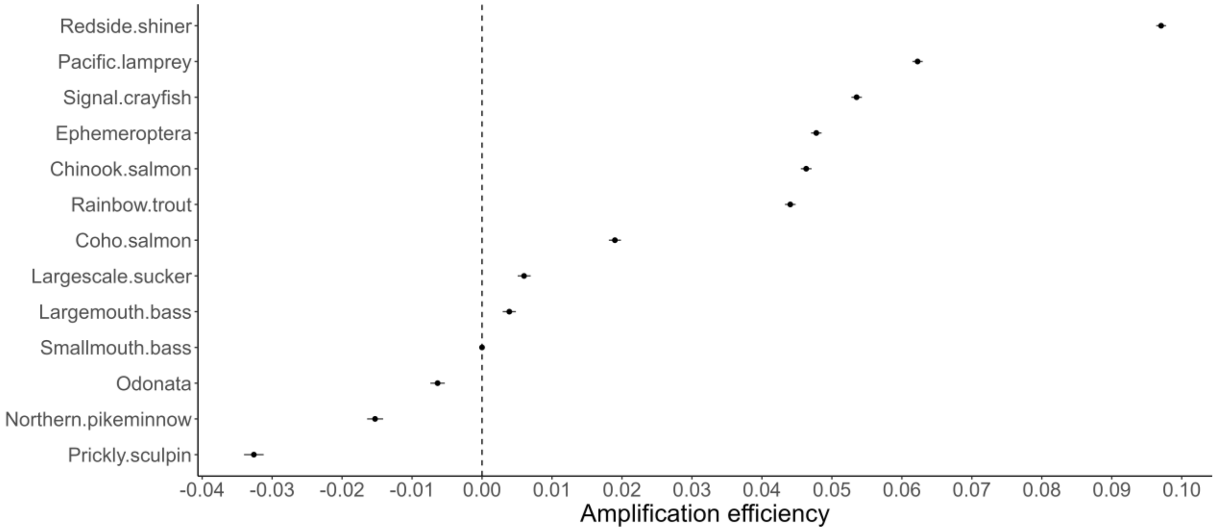

Figure S6. Rock bass mock community posterior amplification efficiency point estimates and 95% confidence intervals.

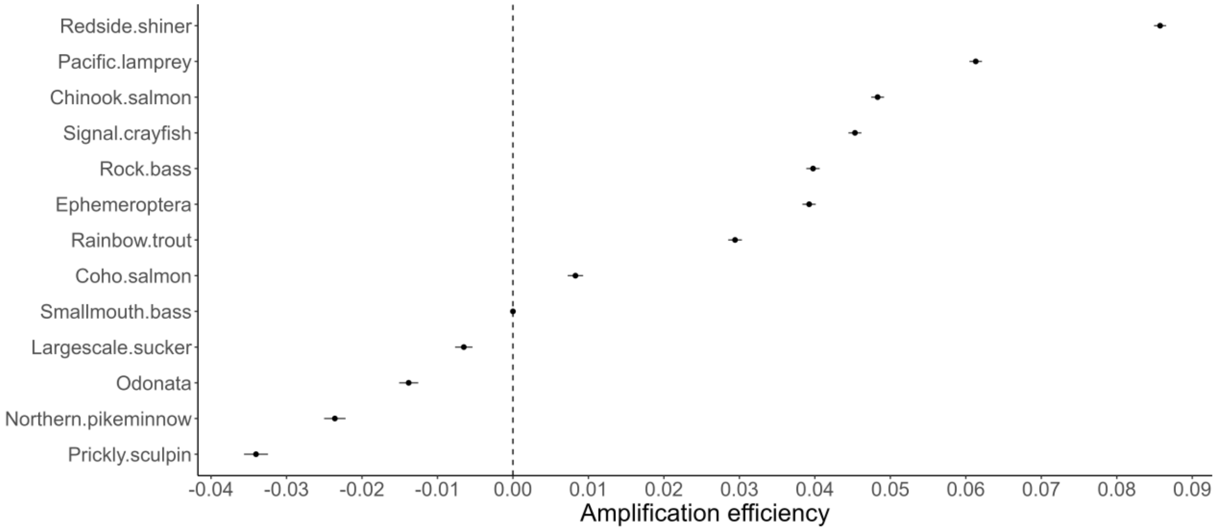

Figure S7. Largemouth bass mock community posterior amplification efficiency point estimates and 95% confidence intervals.

Literature cited

Bolyen, E., J. R. Rideout, M. R. Dillon, N. A. Bokulich, C. C. Abnet, G. A. Al-Ghalith, H. Alexander, E. J. Alm, M. Arumugam, and F. Asnicar. 2019. Reproducible, interactive, scalable and extensible microbiome data science using QIIME 2. Nature biotechnology 37:852–857.

Callahan, B. J., P. J. McMurdie, M. J. Rosen, A. W. Han, A. J. A. Johnson, and S. P. Holmes. 2016. DADA2: High-resolution sample inference from Illumina amplicon data. Nature methods 13:581–583.

Geller, J., C. Meyer, M. Parker, and H. Hawk. 2013. Redesign of PCR primers for mitochondrial cytochrome c oxidase subunit I for marine invertebrates and application in all-taxa biotic surveys. Molecular Ecology Resources 13:851–861.

Leray, M., J. Y. Yang, C. P. Meyer, S. C. Mills, N. Agudelo, V. Ranwez, J. T. Boehm, and R. J. Machida. 2013. A new versatile primer set targeting a short fragment of the mitochondrial COI region for metabarcoding metazoan diversity: application for characterizing coral reef fish gut contents. Frontiers in Zoology 10:34.

Machida, R. J., M. Leray, S.-L. Ho, and N. Knowlton. 2017. Metazoan mitochondrial gene sequence reference datasets for taxonomic assignment of environmental samples. Scientific data 4:1–7.

Martin, M. 2011. Cutadapt removes adapter sequences from high-throughput sequencing reads. EMBnet. journal 17:10–12.

Rognes, T., T. Flouri, B. Nichols, C. Quince, and F. Mahé. 2016. VSEARCH: a versatile open source tool for metagenomics. PeerJ 4:e2584.
